# LoDoPaB-CT, a benchmark dataset for low-dose computed tomography reconstruction

**DOI:** 10.1038/s41597-021-00893-z

**Published:** 2021-04-16

**Authors:** Johannes Leuschner, Maximilian Schmidt, Daniel Otero Baguer, Peter Maass

**Affiliations:** Center for Industrial Mathematics, University of Bremen, Bibliothekstr. 5, 28359 Bremen, Germany

**Keywords:** Applied mathematics, Computed tomography

## Abstract

Deep learning approaches for tomographic image reconstruction have become very effective and have been demonstrated to be competitive in the field. Comparing these approaches is a challenging task as they rely to a great extent on the data and setup used for training. With the Low-Dose Parallel Beam (LoDoPaB)-CT dataset, we provide a comprehensive, open-access database of computed tomography images and simulated low photon count measurements. It is suitable for training and comparing deep learning methods as well as classical reconstruction approaches. The dataset contains over 40000 scan slices from around 800 patients selected from the LIDC/IDRI database. The data selection and simulation setup are described in detail, and the generating script is publicly accessible. In addition, we provide a Python library for simplified access to the dataset and an online reconstruction challenge. Furthermore, the dataset can also be used for transfer learning as well as sparse and limited-angle reconstruction scenarios.

## Abstract (summary)

**Table:** 

Measurement(s)	Low Dose Computed Tomography of the Chest • feature extraction objective
Technology Type(s)	digital curation • image processing technique
Sample Characteristic - Organism	Homo sapiens

Machine-accessible metadata file describing the reported data: 10.6084/m9.figshare.13526360

## Background & Summary

Tomographic image reconstruction is an extensively studied field. One popular imaging modality in clinical and industrial applications is computed tomography (CT). It allows for the non-invasive acquisition of the inside of an object or the human body. The measurements are based on the attenuation of X-ray beams. To obtain the internal distribution of the body from these measurements, an inverse problem must be solved. Traditionally, analytical methods, like filtered back-projection (FBP) or iterative reconstruction (IR) techniques, are used for this task. These methods are the gold standard in the presence of enough high-dose/low-noise measurements. However, as high doses of applied radiation are potentially harmful to the patients, modern scanners aim at reducing the radiation dose. There exist several strategies, but all introduce specific challenges for the reconstruction algorithm, e.g. undersampling or increased noise levels, which require more sophisticated reconstruction methods. The higher the noise or undersampling, the more prior knowledge about the target reconstructions is needed to improve the final quality^1^. Analytical methods are only able to use very limited prior information. Alternatively, machine learning approaches are able to learn underlying distributions and typical image features, which constitute a much larger and flexible prior. Recent image reconstruction approaches involving machine learning, in particular deep learning (DL), have been developed and demonstrated to be very competitive^2–8^.

DL-based approaches benefit strongly from the availability of comprehensive datasets. In the last years, a wide variety of CT data has been published, covering different body parts and scan scenarios. For the training of reconstruction models, the projections (measured data) are crucial but are rarely made available. Recently, Low Dose CT Image and Projection Data (LDCT-and-Projection-data)^9^ was published by investigators from the Mayo Clinic, which include measured normal-dose projection data of 299 patients in the new open DICOM-CT-PD format. The AAPM Low Dose CT Grand Challenge data^10^ includes simulated measurements, featuring 30 different patients. The Finish Inverse Problems Society (FIPS) provides multiple measurements of a walnut^11^ and a lotus root^12^ aimed at sparse data tomography. Recently, Der Sarkissian *et al*.^13^ published cone-beam CT projection data and reconstructions of 42 walnuts. Their dataset is directly aimed at the training and comparison of machine learning methods. In magnetic resonance imaging, fastMRI^14^ with 1600 scans of humans knees is another prominent example.

Other CT datasets focus on the detection and segmentation of special structures like lesions in the reconstructions for the development of computer-aided diagnostic (CAD) methods^15–20^. Therefore, they do not include the projection data. The LIDC/IDRI database^15^, which we use for the ground truth of our dataset (cf. section “Methods”), targets lung nodule detection. FUMPE^16^ contains CT angiography images of 35 subjects for the detection of pulmonary embolisms. KiTS2019^17^ is built around the segmentation of kidney tumours in CT images. The Japanese Society of Radiology Technology (JSRT) database^18^ and the National Lung Screening Trial (NLST) in cooperation with the CT Image Library (CTIL)^19,20^ each contain scans of the lung. These datasets can also be used for the investigation of reconstruction methods by simulating the missing measurements.

Different learned methods have been successfully applied to the task of low-dose reconstruction^7^. However, comparing these approaches is a challenging task since they highly rely on the data and the setup that is used for training. The main goal of this work is to provide a standard dataset that can be used to train and benchmark learned low-dose CT reconstruction methods. To this end, we introduce the Low-Dose Parallel Beam (LoDoPaB)-CT dataset, which uses the public LIDC/IDRI database^15,21,22^ of human chest CT reconstructions. We consider these, in the form of 2D images, to be the so-called ground truth. The projections are created by simulating low photon count CT measurements with a parallel beam scan geometry. Due to the slice-based 2D setup, each of the generated measurements corresponds directly to a ground truth slice. Thus, the reconstruction process can be carried out slice-wise without rebinning^23^, which would have to be applied to the measurements for 3D helical cone-beam geometries commonly used in modern scanners^9^ to allow for the slice-wise use of a 2D reconstruction algorithm. In order to generalise from our dataset to the clinical 3D setup, the effect of rebinning needs to be evaluated. Also, learned algorithms directly targeted at 3D reconstruction should be considered in this case, which at the moment are barely computationally feasible^24^, but presumably outperform 2D reconstruction algorithms applied to rebinned measurements. Despite the generalisation to the 3D case not being straight-forward, our dataset allows to train and compare a large number of approaches applicable to the 2D scenario, which we expect to yield insights for the design of 3D algorithms as well.

Paired samples constitute the most complete training data and could be used for all kinds of learning. In particular, methods that require independent samples from the distributions of images and measurements, or only from one of these distributions, can still make use of the dataset. In total, the dataset features more than 40000 sample pairs from over 800 different patients. This amount of data and variability can be necessary to successfully train deep neural networks^25^. It also qualifies the dataset for transfer learning. In addition, the included measurements can be easily modified for sparse and limited angle scan scenarios.

## Methods

In this section, the considered mathematical model of CT is stated first, followed by a detailed description of the dataset generation. This starts with the LIDC/IDRI database^15^, from which we extract the ground truth reconstructions. Finally, the data processing steps are described, which are also summarised in a semi-formal manner at the end of the section. As a technical reference, the script^26^ used for generation is available online (https://github.com/jleuschn/lodopab_tech_ref).

### Parallel beam CT model

We consider the inverse problem of computed tomography given by

1 $${\mathscr{A}}x+\varepsilon ({\mathscr{A}}x)={y}^{\delta }$$

with:

• ${\mathscr{A}}$ the linear ray transform defined by the scan geometry,

• *x* the unknown interior distribution of the X-ray attenuation coefficient in the body, also called image,

• *ε* a sample from a noise distribution that may depend on the ideal measurement ${\mathscr{A}}$*x*,

• *y*^*δ*^ the noisy CT measurement, also called projections or sinogram.

**Fig. 1:**
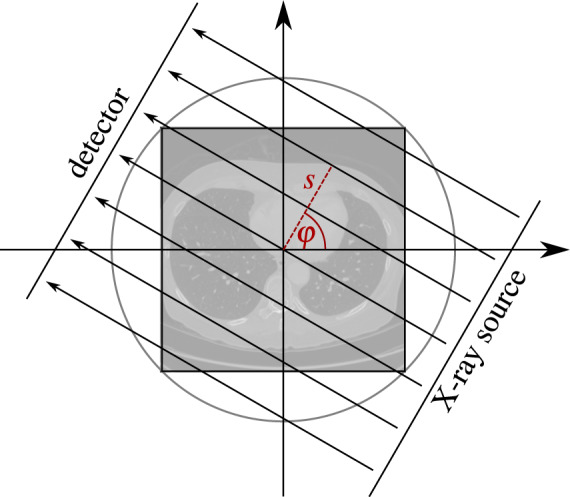
Visualisation^25^ of the parallel beam geometry.

**Fig. 2:**
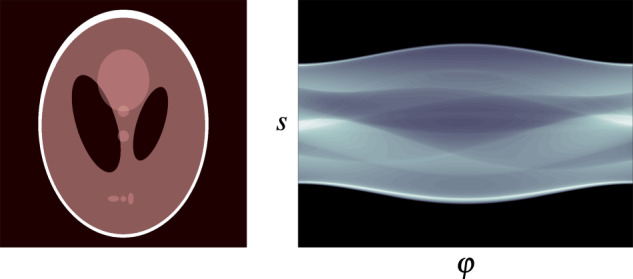
The Shepp-Logan phantom (left) and its corresponding sinogram (right).

More specifically, we choose a two-dimensional parallel beam geometry, for which the ray transform ${\mathscr{A}}$ is the Radon transform^27^. It integrates the values of *x*:${\mathbb{R}}$^2^→${\mathbb{R}}$ fulfilling some regularity conditions (cf. Radon^27^) along the X-ray lines

2 $${L}_{s,\varphi }(t)\,:\,=\,s\omega (\varphi )+t{\omega }^{\perp }(\varphi ),\quad \omega (\varphi )\,:\,=\,\left[\begin{array}{c}{\rm{c}}{\rm{o}}{\rm{s}}(\varphi )\\ {\rm{s}}{\rm{i}}{\rm{n}}(\varphi )\end{array}\right],\quad {\omega }^{\perp }(\varphi )\,:\,=\,\left[\begin{array}{c}-{\rm{s}}{\rm{i}}{\rm{n}}(\varphi )\\ {\rm{c}}{\rm{o}}{\rm{s}}(\varphi )\end{array}\right],$$

for all parameters *s* ∈ ${\mathbb{R}}$ and *φ* ∈ [0, *π*), which denote the distance from the origin and the angle, respectively (cf. Figure 1). In mathematical terms, the image is transformed into a function of (*s*, *φ*),

3 $${\mathscr{A}}x(s,\varphi )\,:\,=\,{\int }_{{\mathbb{R}}}x\left({L}_{s,\varphi }(t)\right){\rm{d}}t,$$

which is called projection, since for each fixed angle *φ* the 2D image *x* is projected onto a line parameterised by *s*, namely the detector. Visualisations of projections as images themselves are called sinograms (cf. Figure 2). The projection relates to the ideal intensity measurements *I*_1_(*s*, *φ*) at the detector according to Beer-Lambert’s law by

4 $${\mathscr{A}}(s,\varphi )=-\,\mathrm{ln}\left(\frac{{I}_{1}\left(s,\varphi \right)}{{I}_{0}}\right)=y\left(s,\varphi \right),$$

where *I*_0_ is the intensity of an unattenuated beam.

In practice, the measured intensities are noisy. The noise can be classified into *quantum noise* and *detector noise*. Quantum noise stems from the process of photon generation, attenuation and detection, which as a whole can be modelled by a Poisson distribution^28^. The detector noise stems from the electronic data acquisition system and is usually assumed to be Gaussian. It would play an important role in ultra-low-dose CT with very small numbers of detected photons^29^ but is neglected in our case. Thus we model the number of detected photons and, by this, the measured intensity ratio with

5 $${\widetilde{N}}_{1}(s,\varphi ) \sim {\rm{Pois}}({N}_{0}\exp (-{\mathscr{A}}x(s,\varphi ))),\quad \frac{{\widetilde{I}}_{1}(s,\varphi )}{{I}_{0}}=\frac{{\widetilde{N}}_{1}(s,\varphi )}{{N}_{0}},$$

where *N*_0_ is the mean photon count without attenuation and Pois(*λ*) denotes the probability distribution defined by

6 $$P(X=k)=\frac{{\lambda }^{k}{e}^{-\lambda }}{k!},\quad k\in {{\mathbb{N}}}_{0}.$$

For practical application, the model needs to be discretised. The forward operator is then a finite-dimensional linear map *A* : ${\mathbb{R}}$^*n*^ → ${\mathbb{R}}$^*m*^, where *n* is the number of image pixels and *m* is the product of the number of detector pixels and the number of angles for which measurements are obtained. The discrete model reads

7 $$Ax+\varepsilon (Ax)={y}^{\delta },\quad \varepsilon (Ax)=-\,Ax-\mathrm{ln}({\widetilde{N}}_{1}/{N}_{0}),\quad {\widetilde{N}}_{1} \sim {\rm{Pois}}({N}_{0}\exp (-Ax)).$$

Here, Pois(*λ*) denotes the joint distribution of *m* Poisson distributed observations with parameters *λ*_1_, …, *λ*_*m*_, respectively. Note that since the negative logarithm is applied to the observations, the noisy post-log values *y*^*δ*^ do not follow a Poisson distribution but the distribution resulting from this log-transformation. However, taking the negative logarithm is required to obtain the linear model and therefore is most commonly applied as a preprocessing step. For our dataset, we consider post-log values by default.

The Radon transform is a linear and compact operator. Therefore, the continuous inverse problem of CT is mildly ill-posed in the sense of Nashed^30,31^. This means that small variations in the measurements can lead to significant differences in the reconstruction (unstable inversion). While the discretised inverse problem is not ill-posed, it is typically ill-conditioned^28^, which leads to artefacts in reconstructions obtained by direct inversion from noisy measurements.

For our discrete simulation setting, we use the described model with following dimensions and parameters:

• Image resolution of 362 px × 362 px on a domain of size 26 cm × 26 cm.

• 513 equidistant detector bins *s* spanning the image diameter.

• 1000 equidistant angles *φ* between 0 and *π*.

• Mean photon count per detector bin without attenuation *N*_0_ = 4096.

### LIDC/IDRI database and data selection

The Lung Image Database Consortium (LIDC) and Image Database Resource Initiative (IDRI) published the LIDC/IDRI database^15,21,22^ to support the development of CAD methods for the detection of lung nodules. The dataset consists of 1018 helical thoracic CT scans of 1010 individuals. Seven academic centres and eight medical imaging companies collaborated for the creation of the database. As a result, the data is heterogeneous with respect to the technical parameters and scanner models.

**Fig. 3:**
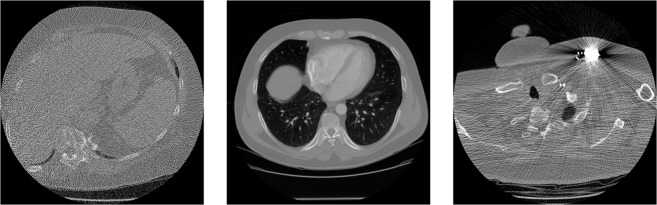
Scans from the LIDC/IDRI database^15^ with poor quality, good quality and an artefact. The shown HU window is [−1024, 1023].

Both standard-dose and lower-dose scans are part of the dataset. Tube peak voltages range from 120 kV to 140 kV and tube current from 40 mA to 627 mA with a mean of 222.1 mA. Labels for the lung nodules were created by a group of 12 radiologists in a two-phase process. The image reconstruction was performed with different filters, depending on the manufacturer of the scanner. Figure 3 shows examples of the provided reconstructions. The LIDC/IDRI database is freely available from The Cancer Imaging Archive (TCIA)^22^. It is published under the Creative Commons Attribution 3.0 Unported License (https://creativecommons.org/licenses/by/3.0/).

The LoDoPaB-CT dataset is based on the LIDC/IDRI scans. Our dataset is intended for the evaluation of reconstruction methods in a low-dose setting. Therefore, we simulate the projection data, which is not included in the LIDC/IDRI database. In order to enable a fair comparison with good ground truth, scans that are too noisy were removed in a manual selection process (cf. section “Technical Validation”). Additional scans were excluded due to their geometric properties, namely an image size different from 512 px × 512 px, a too small area of valid pixel values (cf. subsection “Ground truth image extraction” below), or a different patient orientation. The complete lists of excluded scan series are given in file series_list.json in the technical reference repository^26^. In the end, 812 patients remain in the LoDoPaB-CT dataset.

The dataset is split into four parts: three parts for training, validation and testing, respectively, and a “challenge” part reserved for the LoDoPaB-CT Challenge (https://lodopab.grand-challenge.org/). Each part contains scans from a distinct set of patients, as we want to study the case of learned reconstructors being applied to patients that are not known from training. The training set features scans from 632 patients, while the other parts contain scans from 60 patients each. Every scan contains multiple slices (2D images) for different *z*-positions, of which only a subset is included. The amount of extracted slices depends on the slice thickness obtained from the metadata. As slices with small distances are similar, they may not provide much additional information while increasing the chances to overfit. The distances of the extracted slices are larger than 5.0 mm for >45% and larger than 2.5 mm for >75% of the slices. In total, the dataset contains 35820 training images, 3522 validation images, 3553 test images and 3678 challenge images.

#### Remark

We propose to use our default dataset split, as it allows for a fair comparison with other methods that use the same split. However, users are free to remix or re-split the dataset parts. For this purpose, randomised patient IDs are provided, i.e., the same random ID is given for all slices obtained from one patient. Thus, when creating custom splits it can be regulated whether—and to what extent—data from the same patients are contained in different splits.

### Ground truth image extraction

First, each image is cropped to the central rectangle of 362 px × 362 px. This is done because most of the images contain (approximately) circle-shaped reconstructions with a diameter of 512 px (cf. Figure 3). After the crop, the image only contains pixels that lie inside this circle, which avoids value jumps occurring at the border of the circle. While this yields natural ground truth images, we need to point out that the cropped images, in general, do not show the full subject but some interior part. Hence, it is unlikely for methods trained with this dataset to perform well on full-subject measurements.

For some scan series, the circle is subject to a geometric transformation either shrinking or expanding the circle in some directions. In particular, for a few scan series, the circle is shrunk such that it is smaller than the cropped rectangle. We exclude these series, i.e. those with patient IDs 0004, 0032, 0102, 0116, 0120, 0289, 0368, 0418, 0541, 0798, 0926, 0972 and 1000, from our dataset, which allows to crop all included images consistently to 362 px × 362 px.

The integer Hounsfield unit (HU) values obtained from the DICOM files are dequantised by adding uniform noise from the interval [0, 1). By adding this noise, the discrete distribution of stored values is transformed into a continuous distribution (up to the floating-point precision), which is a common assumption of image models. For example, the meaningful evaluation of densities learned by generative networks requires dequantization^32^, which in some works^33^ is more refined than the uniform dequantization applied to the HU values in our dataset.

In the next step, the linear attenuations *μ* are computed from the dequantised HU values using the definition of the HU,

8 $${\rm{HU}}=1000\frac{\mu -{\mu }_{{\rm{water}}}}{{\mu }_{{\rm{water}}}-{\mu }_{{\rm{air}}}}\quad \quad \iff \quad \quad \mu ={\rm{HU}}\frac{{\mu }_{{\rm{water}}}-{\mu }_{{\rm{air}}}}{1000}+{\mu }_{{\rm{water}}},$$

where we use the linear attenuation coefficients

9 $${\mu }_{{\rm{water}}}=20/{\rm{m}},\quad {\mu }_{{\rm{air}}}=0.02/{\rm{m}},$$

which approximately correspond to an X-ray energy of 60 keV^34^. Finally, the *μ*-values are normalised into [0, 1] by dividing by

10 $${\mu }_{{\rm{\max }}}=3071\frac{{\mu }_{{\rm{water}}}-{\mu }_{{\rm{air}}}}{1000}+{\mu }_{{\rm{water}}}=81.35858/{\rm{m}},$$

which corresponds to the largest HU value that can be represented with the standard 12-bit encoding, i.e. (2^12^–1–1024)HU = 3071 HU, followed by the clipping of all values into the range [0, 1],

11 $$\widehat{\mu }={\rm{clip}}(\mu /{\mu }_{{\rm{\max }}},[0,1])=\left\{\begin{array}{ll}0 & ,\mu /{\mu }_{{\rm{\max }}}\le 0\\ \mu /{\mu }_{{\rm{\max }}} & ,0 < \mu /{\mu }_{{\rm{\max }}}\le 1\\ 1 & ,1 < \mu /{\mu }_{{\rm{\max }}}\end{array}\right..$$

The Eqs. (8) and (11) are applied pixel-wise to the images.

### Projection data generation

To simulate the measurements based on the virtual ground truth images, the main step is to apply the forward operator, which is the ray transform (Radon transform in 2D) for CT. For this task we utilise the Operator Discretization Library^35^ (ODL) with the ‘astra_cpu’ backend^36^.

#### Remark

We choose ‘astra_cpu’ over the usually favoured ‘astra_cuda’ because of small inaccuracies observed in the sinograms when using ‘astra_cuda’, specifically at angles 0, $\frac{\pi }{2}$ and *π* and detector positions $-1/\sqrt{2}\frac{l}{2}$ and $1/\sqrt{2}\frac{l}{2}$ with *l* being the length of the detector. The used version is astra-toolbox==1.8.3 on Python 3.6. The tested CUDA version is 9.1 combined with cudatoolkit==8.0.

In order to avoid “committing the inverse crime”^37^, which, in our scenario, would be to use the same discrete model both for simulation and reconstruction, we use a higher resolution for the simulation. Otherwise, good performance of reconstructors for the specific resolution of this dataset (362 px × 362 px) could also stem from the properties of the specific discretised problem, rather than from good inversion of the analytical model. We use bilinear interpolation for the upscaling of the virtual ground truth from 362 px × 362 px to 1000 px × 1000 px.

**Fig. 4:**
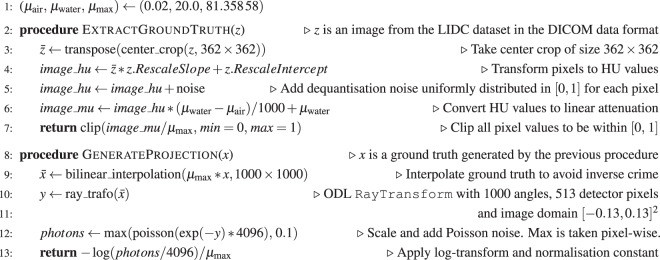
Data generation algorithm.

The non-normalised, upscaled image is projected by the ray transform. Based on this projection, *Ax*, the measured photon counts $\mathop{N}\limits^{ \sim }$_1_ are sampled according to Eq. (7). The sampling in some cases yields photon counts of zero, which we then replace by photon counts of 0.1. Hereby strictly positive values are ensured, which is a prerequisite for the log-transform in the next step (cf. Wang *et al*.^38^). The negative logarithm of the photon counts quotient max(0, 1, $\mathop{N}\limits^{ \sim }$_1_)*N*_0_ is taken, resulting in the post-log measurements *y*^*δ*^ according to Eq. (7) (up to the 0.1 photon count approximation). Finally, *y*^*δ*^ is divided by *μ*_max_ to match the normalised ground truth images. A summary of all steps can be found in Fig. 4 (Data generation algorithm).

#### Remark

Although the linear model obtained by the log-transform is easier to study, in some cases pre-log models are more accurate. See Fu *et al*.^29^ for a detailed comparison. For applying a pre-log method, the stored observation data $\widehat{y}={y}^{\delta }/{\mu }_{{\rm{\max }}}$ must be back-transformed by ${\rm{e}}{\rm{x}}{\rm{p}}(-{\mu }_{{\rm{\max }}}\cdot \widehat{y})$. To create physically consistent data pairs, the ground truth images should then be multiplied with *μ*_max_, too.

#### Remark

Note that the minimum photon count of 0.1 can be adapted subsequently. This is most easily done by filtering out the highest observation values and replacing them with −log(*ε*_0_/4096)/*μ*_max_, where *ε*_0_ is the new minimum photon count.

## Data Records

The LoDoPaB-CT dataset is published as open access on Zenodo (https://zenodo.org) in two repositories. The main data repository^39^ (10.5281/zenodo.3384092) has a size of around 55GB and contains observations and ground truth data of the train, validation and test set. For each subset, represented by *, the following files are included:

- CSV files patient_ids_rand_*.csv include randomised patient IDs of the samples. The patient IDs of the train, validation and test parts are integers in the range of 0–631, 632–691 and 692–751, respectively. The ID of each sample is stored in a single row.
- Zip archives ground_truth_*.zip contain HDF5^40^ files of the ground truth reconstructions.
- Zip archives observation_*.zip contain HDF5 files of the simulated low-dose measurements.
- Each HDF5 file contains one HDF5 dataset named data, that provides several samples (128 except for the last file in each ZIP file). For example, the *n*-th training sample pair is stored in the HDF5 files observation_train_%03d.hdf5 and ground_truth_train_%03d.hdf5 where the placeholder %03d is floor (*n*/128). Within these HDF5 files, the observation or ground truth is stored at entry (*n* mod 128) of the HDF5 dataset data.
  The second repository^41^ for the challenge data (10.5281/zenodo.3874937) consists of a single zip archive:
- observation_challenge.zip contains HDF5 files of the simulated low-dose measurements.

The structure inside the HDF5 files is the same as in the main repository.

## Technical Validation

### Ground truth & data selection

Creating high-quality ground truth images for tomographic image reconstruction is a challenging and time-consuming task. In computed tomography, one option is to cut open the object after the scan or use 3D printing^42^, whereby the digital template of the object is the reference. In general, this also involves high radiation doses and many scanning angles. This combination makes it even harder to generate ground truth images for medical applications.

For low-dose CT reconstruction models, the primary goal is to match the normal-dose reconstruction quality of methods currently in use. Therefore, normal-dose reconstructions from classical methods, e.g. filtered back-projection, are an adequate choice as ground truth. This simplifies the process considerably.

The ground truth CT reconstructions of LoDoPaB-CT are taken from the established and well-documented LIDC/IDRI database. An independent visual inspection of one 2D slice per scan was performed by three of the authors. Figure 3 shows three examples of such slices. A five-star rating system was used to evaluate the image quality and remove noisy ground truth data, like the first slice in Fig. 3. Scans with artefacts, e.g. from photon starvation due to dense material (cf. Figure 3 (right)), were in general not removed, as the artefacts only affect a few slices of the whole scan. The slice in the middle of Fig. 3 represents an ideal ground truth. The following procedure was then used to exclude scans based on their rating:

1. Centring of the ratings from each evaluator around the value 3.
2. Calculation of the mean rating and the variance for each looked at 2D slice.
3. For a variance <1, the mean was used as the rating score. Otherwise, the scan is evaluated by all three authors together.
4. All scans with a rating ≤2 are excluded from the dataset.

These excluded scans are listed at key “series_excluded_manual_low_q_filter” in file series_list.json in the technical reference repository^26^.

### Reference reconstructions & quantitative results

To validate the usability of the proposed dataset for machine learning approaches, we provide reference reconstructions and quantitative results for the standard filtered back-projection (FBP) and a learned post-processing method (FBP + U-Net). FBP is a widely used analytical reconstruction technique (cf. Buzug^28^ for an introduction). If the measurements are noisy (due to the low dose), FBP reconstructions tend to include streaking artefacts. A typical approach to overcome this problem is to apply some post-processing such as denoising. Recent works^3,4,8^ have successfully used convolutional neural networks, such as the U-Net^43^. The idea is to train a neural network to create clean reconstructions out of the noisy FBP results.

**Fig. 5:**
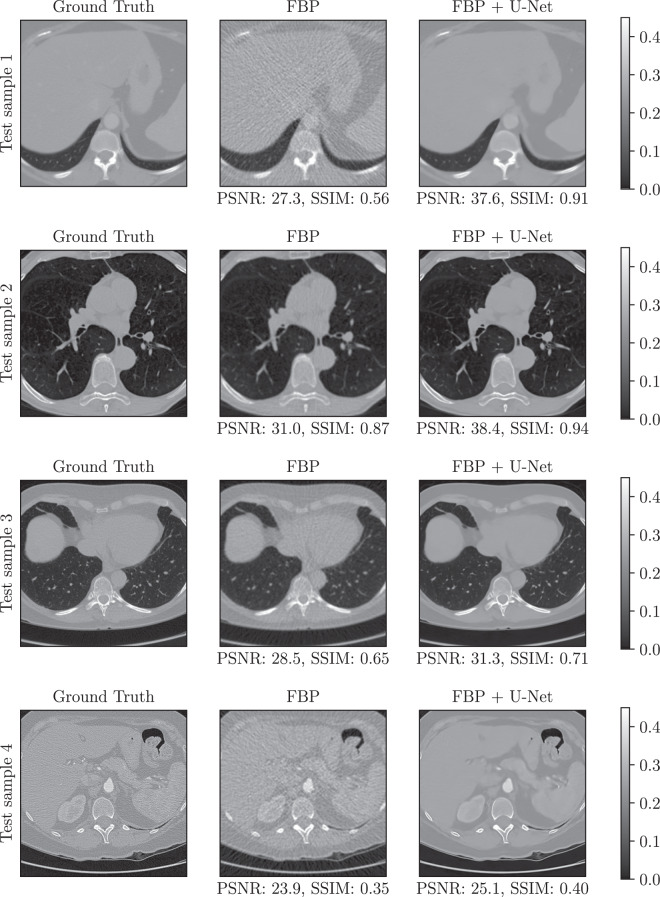
Different baseline reconstructions from the FBP and FBP + U-Net methods. The ground truth images are part of the LoDoPaB-CT test set. The window [0, 0.45] corresponds to a HU range of ≈[−1001, 831].

In this initial study, for the FBP, we used the Hann filter with a frequency scaling of 0.641. We selected these parameters based on the performance over the first 100 samples of the validation dataset. For the post-processing approach (FBP + U-Net), we used a U-Net-like architecture with 5 scales. We trained it using the proposed dataset by minimising the mean squared error loss with the Adam algorithm^44^ for a maximum of 250 epochs with batch size 32. Additionally, we used an initial learning rate of 10^−3^, decayed using cosine annealing until 10^−4^. The model with the highest mean peak signal-to-noise ratio (PSNR) on the validation set was selected from the models obtained during training. Sample reconstructions are shown in Fig. 5.

Table 1 depicts the obtained results in terms of the peak signal-to-noise ratio (PSNR) and structural similarity^45^ (SSIM) metrics (cf. “Evaluation practice” in the next section for a detailed explanation). As it can be observed, the post-processing approach, which was trained using the proposed dataset, outperforms the classical FBP reconstructions by a margin of 5 dB. This demonstrates that the dataset indeed contains valuable data ready to be used for training machine learning methods to obtain CT reconstructions with higher quality than the standard methods.

**Table 1:** Baseline performance.

	training set		validation set		test set	
	PSNR (dB)	SSIM	PSNR (dB)	SSIM	PSNR (dB)	SSIM
FBP	30.45 ± 2.65	0.7415 ± 0.1314	30.75 ± 2.52	0.7577 ± 0.1231	30.52 ± 3.10	0.7372 ± 0.1467
FBP + U-Net	36.17 ± 3.75	0.8623 ± 0.1228	36.74 ± 3.28	0.8819 ± 0.1017	35.84 ± 4.59	0.8443 ± 0.1501

Values are the mean and standard deviation over all samples.

## Usage Notes

### Download & Easy access

The whole LoDoPaB-CT dataset^39,41^ can be downloaded directly from the Zenodo website. However, we recommend the Python library DIV*α*${\mathscr{l}}$^46^ (https://github.com/jleuschn/dival) for easy access of the dataset. The library includes specific functionalities for the interaction with the provided dataset.

#### Remark

Access to the dataset on Zenodo might be restricted or slow in some regions of the world. In this case please contact one of the corresponding authors to get an alternative download option.

DIV*α*${\mathscr{l}}$ is also available through the package index PyPI (https://pypi.org/project/dival). With the library, the dataset is automatically downloaded, checked for corruption and ready for use within two lines of Python code:

**from** dival **import** get_standard_dataset

dataset = get_standard_dataset(‘lodopab’).

#### Remark

When loading the dataset using DIV*α*${\mathscr{l}}$, an ODL^35^ RayTransform implementing the forward operator is created. This requires a backend, the default being ‘astra_cuda’, which requires both the astra toolbox^36^ and CUDA to be available. If either is unavailable, a different backend (‘astra_cpu’ or ‘skimage’) must be selected by keyword argument impl.

In addition, DIV*α*${\mathscr{l}}$ offers multiple options to work with the LoDoPaB-CT dataset:

- Access the train, validation and test subset and draw a specific number of samples.
- Sort the data by the patient ids.
- Use the pre-log or post-log data (cf. projection data generation in the “Methods” section).
- Evaluate the reconstruction performance.
- Compare with pre-trained standard reconstruction models.

### Evaluation practice

Since ground truth data is provided in the dataset, we recommend using so-called full-reference methods for the evaluation. The peak signal-to-noise ratio (PSNR) and the structural similarity^45^ (SSIM) are two standard image quality metrics often used in CT applications^42,47^. While the PSNR calculates pixel-wise intensity comparisons between ground truth and reconstruction, SSIM captures structural distortions.

#### Peak signal-to-noise ratio

The PSNR expresses the ratio between the maximum possible image intensity and the distorting noise, measured by the mean squared error (MSE),

12 $${\rm{PSNR}}\left(\widetilde{x},x\right)\,:\,=\,10{log}_{10}\left(\frac{{\rm{m}}{\rm{a}}{{\rm{x}}}_{x}^{2}}{{\rm{MSE}}\left(\widetilde{x},x\right)}\right),\quad {\rm{MSE}}\left(\widetilde{x},x\right)\,:\,=\,\frac{1}{n}\mathop{\sum }\limits_{i=1}^{n}{\left|{\widetilde{x}}_{i}-{x}_{i}\right|}^{2}.$$

Here *x* is the ground truth image and $\widetilde{x}$ the reconstruction. Higher PSNR values are an indication of a better reconstruction. We recommend choosing max_*x*_ = max(*x*) − min(*x*), i.e. the difference between the highest and lowest entry in *x*, instead of the maximum possible intensity, since the reference value of 3071HU is far from the most common values. Otherwise, the results can often be too optimistic.

#### Structural similarity

Based on assumptions about the human visual perception, SSIM compares the overall image structure of ground truth and reconstruction. Results lie in the range [0, 1], with higher values being better. The SSIM is computed through a sliding window at *M* locations

13 $${\rm{SSIM}}\left(\widetilde{x},x\,\right)\,:\,=\,\frac{1}{M}\mathop{\sum }\limits_{j=1}^{M}\frac{\left(2{\widetilde{\mu }}_{j}{\mu }_{j}+{C}_{1}\right)\left(2{\Sigma }_{j}+{C}_{2}\right)}{\left({\widetilde{\mu }}_{j}^{2}+{\mu }_{j}^{2}+{C}_{1}\right)\left({\widetilde{\sigma }}_{j}^{2}+{\sigma }_{j}^{2}+{C}_{2}\right)},$$

where ${\widetilde{\mu }}_{j}$ and ${\mu }_{j}$ are the average pixel intensities, ${\widetilde{\sigma }}_{j}$ and *σ*_*j*_ the variances and ${\Sigma }_{j}$ the covariance of $\widetilde{x}$ and *x* at the *j*-th local window. Constants ${C}_{1}={({K}_{1}L)}^{2}$ and ${C}_{2}={({K}_{2}L)}^{2}$ stabilise the division. Following Wang *et al*.^45^ we choose *K*_1_ = 0.01 and *K*_2_ = 0.03 for the technical validation in this paper. The window size is 7 × 7 and *L* = max(*x*) − min(*x*).

#### Test & challenge set

The test data is the advised subset for offline model evaluation. To guarantee a fair comparison, the data should be in no way involved in the training process or hyperparameter selection of the model. We recommend using the whole test set and select the above-mentioned parameters for PSNR and SSIM. Deviations from this setting should be mentioned.

In addition, a challenge set without ground truth images is provided. We encourage users to submit their challenge reconstructions to the evaluation website (https://lodopab.grand-challenge.org/). All methods are assessed under the same conditions and with the same metrics. The performance can be directly compared with other methods on a public leaderboard. Therefore, we recommend to report performance measures on the challenge set for publications that use the LoDoPaB-CT dataset without modifications, in addition to any evaluations on the test set. In accordance with the Biomedical Image Analysis (BIAS) guidelines^48^, more information about the challenge can be found on the aforementioned website.

### Further usage

#### Scan scenarios

The provided measurements and simulation scripts can easily be modified to cover different scan scenarios:

- Limited and sparse-angle problems can be created by loading a subset of the projection data, e.g. a sparser setup with 200 angles was already used by Baguer *et al*.^25^.
- Super-resolution experiments can be mimicked, by artificially binning the projection data into larger pixels.
- To study lower or higher photon counts, the dataset can be re-simulated with a different value of *N*_0_ (e.g. using resimulate_observations.py^26^ by changing the value of PHOTONS_PER_PIXEL).

The provided reconstructions can still be used as ground truth for all listed scenarios.

#### Transfer learning

Transfer learning is a popular approach to boost the performance of machine learning models on smaller datasets. The idea is to first train the model on a different, comprehensive data collection. Afterwards, the determined parameters are used as an initial guess for fine-tuning the model on the smaller one. In general, the goal is to learn to process low-level features, e.g. edges in images, from the comprehensive dataset. The adaption to specific high-level features is then performed on the smaller dataset. For imaging applications, the ImageNet database^49^, with over 14 million natural images, is frequently used in this role. The applications range from image classification^50^ to other domains like audio data^51^.

Transfer learning has also been successfully applied to CT reconstruction tasks. This includes training on different scan scenarios^52,53^, e.g. a different number of angles, as well as first training on 2D data and continuing on 3D data^54^. He *et al*.^55^ simulated parallel beam measurements on some of the natural images contained in ImageNet. Subsequently, the training was continued on CT images from the Mayo Clinic^10^. LoDoPaB-CT, or parts of the dataset, can be used in similar roles for transfer learning. Additionally, the ground truth data from real thoracic CT scans may be advantageous for similar CT reconstruction tasks compared to random natural images from ImageNet^56^.

Nonetheless, we advise the user to check the applicability for their specific use case and reconstruction model. Re-simulation or other changes to the LoDoPaB-CT dataset might be needed, especially for datasets with different scan geometries. Additionally, simulated data can not capture all aspects of real-world measurements and therefore cause reconstruction errors. For a comprehensive study on the benefits and challenges of transfer learning for medical imaging, we refer the reader to the publication by Raghu *et al*.^56^.

#### Remark

An example for a simulation script with a fan beam geometry on the ground truth data can be found in the DIV*a*${\mathscr{l}}$^46^ library: dival/examples/ct_simulate_fan_beam_from_lodopab_ground_truth.py.

### Limits of the dataset

The LoDoPaB-CT dataset is designed for a methodological comparison of CT reconstruction methods on a simulated low-dose parallel beam setting. The focus is on how a model deals with the challenges that arise from low photon count measurements to match the quality of normal-dose images. Of course, this represents only one aspect of many for the application in real-world scenarios. Therefore, results achieved on LoDoPaB-CT might not completely reflect the performance on real medical data. The following limits of the dataset should be considered when evaluating and comparing results:

- The simulation uses the Radon transform and Poisson noise. Real measurements can be influenced by additional physical effects, like scattering.
- Modern CT machines use advanced scanning geometries, like helical fan beam or cone beam. Specific challenges for the reconstruction can arise compared to parallel beam measurements (cf. Buzug^28^).
- In general, the goal is to reconstruct a whole 3D subject and not just a single 2D slice. Reconstruction methods might benefit from additional spacial information. On the other hand, requirements on memory and compute power can be higher for methods that reconstruct 3D volumes directly.
- Image metrics, e.g. PSNR and SSIM, cannot express and cover all aspects of high-quality CT reconstruction. An additional assessment by experts in the field can be beneficial.
- The ground truth images are based on reconstructions from normal-dose medical scans. As such, they can contain noise and artefacts. The measurements are created from this “noisy” ground truth. Therefore, a perfect reconstruction model would re-create the imperfections. Approaches that are designed to remove them can score lower PSNR and SSIM values, although their reconstruction quality might be higher.
- A crop to a region of interest is used for the ground truth images (cf. “Ground truth image extraction”). Hence, the results for full-subject measurements can be different.

## Data Availability

Python scripts^26^ for the simulation setup and the creation of the dataset are publicly available on Github (https://github.com/jleuschn/lodopab_tech_ref). They make use of the ASTRA Toolbox^36^ (version 1.8.3) and the Operator Discretization Library^35^ (ODL, version ≥0.7.0). In addition, the ground truth reconstructions from the LIDC/IDRI database^21^ are needed for the simulation process. A sample data split into training, validation, test and challenge part is also provided. It differs from the one used for the creation of this dataset in order to keep the ground truth data of the challenge set undisclosed. The random seeds used in the scripts are modified for the same reason. The authors acknowledge the National Cancer Institute and the Foundation for the National Institutes of Health, and their critical role in the creation of the free publicly available LIDC/IDRI database used in this study.
